# Radiologic evaluation of compensatory lung growth using computed tomography by comparison with histological data from a large animal model

**DOI:** 10.1038/s41598-022-06398-y

**Published:** 2022-02-15

**Authors:** Keiji Ohata, Toyofumi F. Chen-Yoshikawa, Masatsugu Hamaji, Takeshi Kubo, Tatsuo Nakamura, Hiroshi Date

**Affiliations:** 1 Department of Thoracic Surgery, Kyoto University Graduate School of Medicine, 54 Shogoin Kawahara-cho, Sakyo-ku, Kyoto, 606-8507 Japan; 2 Department of Thoracic Surgery, Nagoya University Graduate School of Medicine, Aichi, Japan; 3 Department of Diagnostic Imaging and Nuclear Medicine, Kyoto University Graduate School of Medicine, Kyoto, Japan; 4 Department of Radiology, Tenri Hospital, Nara, Japan; 5 Department of Bioartificial Organs Institute for Frontier Medical Sciences, Kyoto University, Kyoto, Japan

**Keywords:** Computational biology and bioinformatics, Anatomy, Medical research

## Abstract

Non-invasive analysis using computed tomography (CT) data may be a promising candidate to evaluate neo-alveolarization in adult lungs following lung resection. This study evaluates and compares the validity of CT analysis with histologic morphometry for compensatory lung growth in a large animal model. We calculated the radiologic tissue volume and the radiologic lung weight from CT data taken at 1, 3, and 6 months post-surgery on 15 male beagle dogs that had a right thoractotomy, bilobectomy, or pneumonectomy (n = 5 in each group). Results were analyzed using one-way ANOVA and were subsequently compared to histologic findings of tissue samples at 6 months post-surgery using Pearson’s correlation. An increase in radiologic tissue volume and radiologic lung weight was identified, which was positively correlated with histologic lung parenchymal amounts (correlation coefficient = 0.955 and 0.934, respectively, *p* < 0.001). Histology of lung specimens at 6 months post-surgery revealed an increase in the tissue amount in both Bilobectomy and Peumonectomy groups, which was consistent with compensatory lung growth. Radiologic tissue volume and radiologic lung weight reflected compensatory lung growth following lung resection. Radiologic assessment using CT data can be a promising clinical modality to evaluate postoperative lung growth.

## Introduction

Formation of new alveoli (neo-alveolarization) in the adult lungs following lung resection remains a controversial topic. It is generally believed that the process of increasing alveolar numbers is completed until approximately 8 years old in humans, and that this number remains constant following completion^1,2^. However, Butler et al. have reported the occurrence of alveolar regeneration in the residual lung of a 33-year-old female following right pneumonectomy using hyperpolarized helium-3 magnetic resonance imaging^3^. This in turn suggests that neo-alveolarization can occur even in adult lungs, if the appropriate conditions are met. Despite the fact that further detailed studies on human lungs are warranted so as to validate this phenomenon, it is very difficult to perform frequent histologic evaluations with lung biopsies in clinical practice, especially in postoperative states. Therefore, it is essential to develop novel, non-invasive and analytic methodologies.

Radiologic evaluation using computed tomography (CT) images is a candidate for the effective evaluation of neo-alveolarization. CT examination is one of the most frequently performed examinations in current clinical practice that can assess postoperative lung function in an efficient manner. Its highly improved spatial resolution due to recent technological advancements enables qualitative assessment of lung architectures^4^. In fact, this imaging method has been already utilized to assess the severity of emphysema^5^, idiopathic pulmonary fibrosis^6^, and acute respiratory distress syndrome^7^. Hsia et al. suggested the effectiveness of “*tissue volume*” term in large animal studies, and, correspondingly, we proposed the term “*radiologic lung weight*” in human data analysis as calculated values using CT images in compensatory lung growth^8–12^. However, these radiologic measurements are still not universally accepted in regenerative studies. Consequently, the aim of this study is to evaluate the validity of CT-derived “radiologic tissue volume” and “radiologic lung weight” prospectively by means of comparison with histologic morphometry in a large animal model.

## Results

### Radiologic lung volumetry and measurement of lung tissue amount

#### Lung volumetry in radiologic analysis

**Figure 1:**
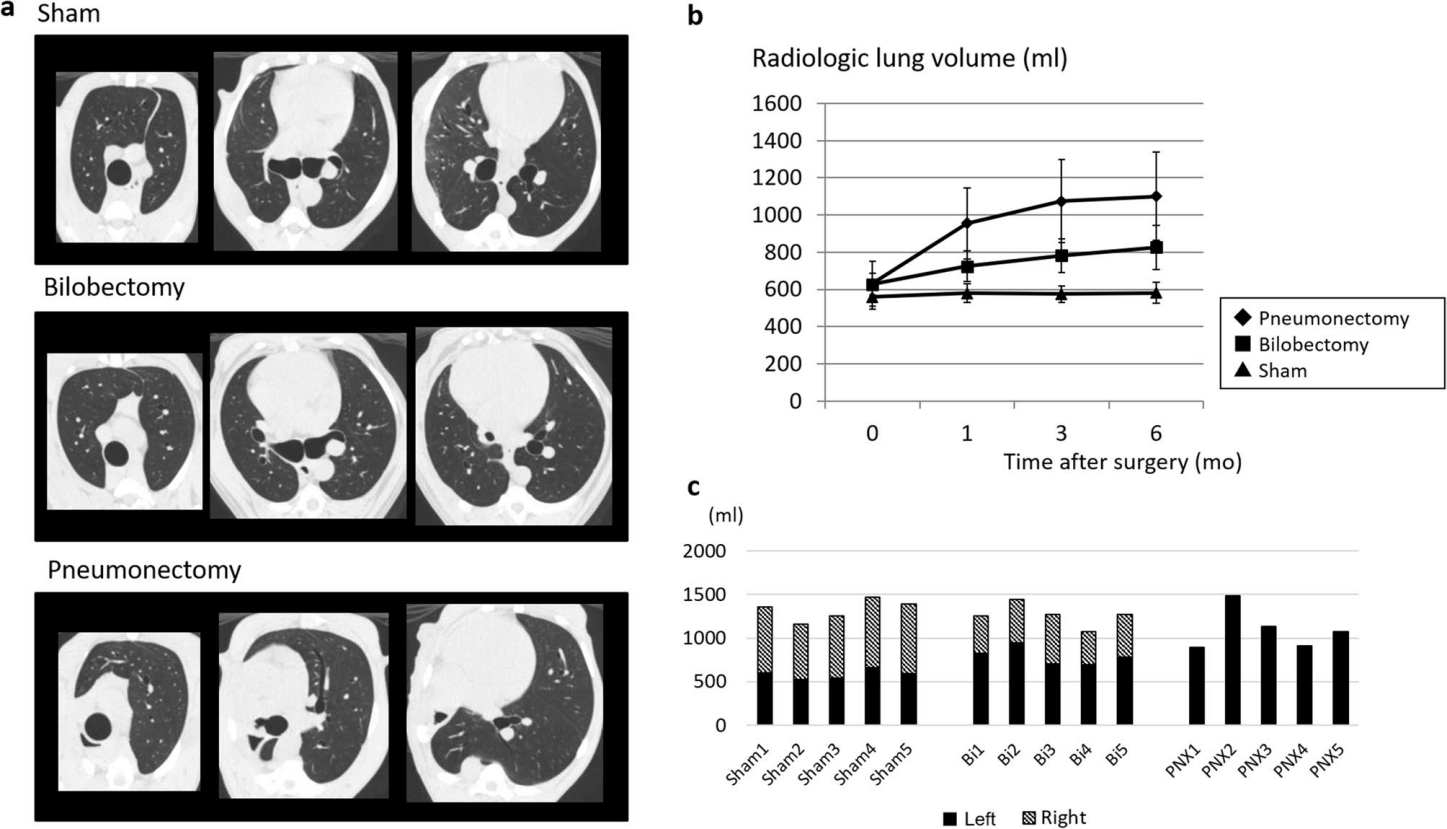
(**a**) Representative computed tomography (CT) images 6 months after surgery of the Pneumonectomy, Bilobectomy, and Sham groups. (Window level: − 600, window width: 1500. Reconstruction kernel: FC13. Reconstructed slice thickness: 1 mm.) (**b**) The time course of lung volume measured from CT images. (**c**) The values of radiologic lung volume of the left and right lungs in each canine at 6 months after surgery. Bi: Bilobectomy, PNX: Pneumonectomy.

None of the animals included in this study had surgery-related death or comorbidity. The representative images of each group and the time course of lung volume measured from the CT data are shown in Fig. 1a and b, respectively. Lung volumes at 6 months post-surgery in the Sham, Bilobectomy, and Pneumonectomy groups were 582.5 ± 55.3 ml, 791.8 ± 102.4 ml, and 1100.8 ± 238.1 ml, respectively. Furthermore, the increase rates were 4.7 ± 7.5%, 26.2 ± 11.3%, and 73.5 ± 5.2%, respectively. The rates of increase were significantly higher in the Pneumonectomy group than in the other two groups (*p* < 0.001, each), and the increase rates in the Bilobectomy group were significantly higher than that in the Sham group (*p* = 0.004). For comparison, the values of radiologic lung volume of the left and right lungs in each canine 6 months after surgery are presented in Fig. 1c.

#### Lung tissue amount in radiologic analysis

##### Radiologic tissue volume

**Figure 2:**
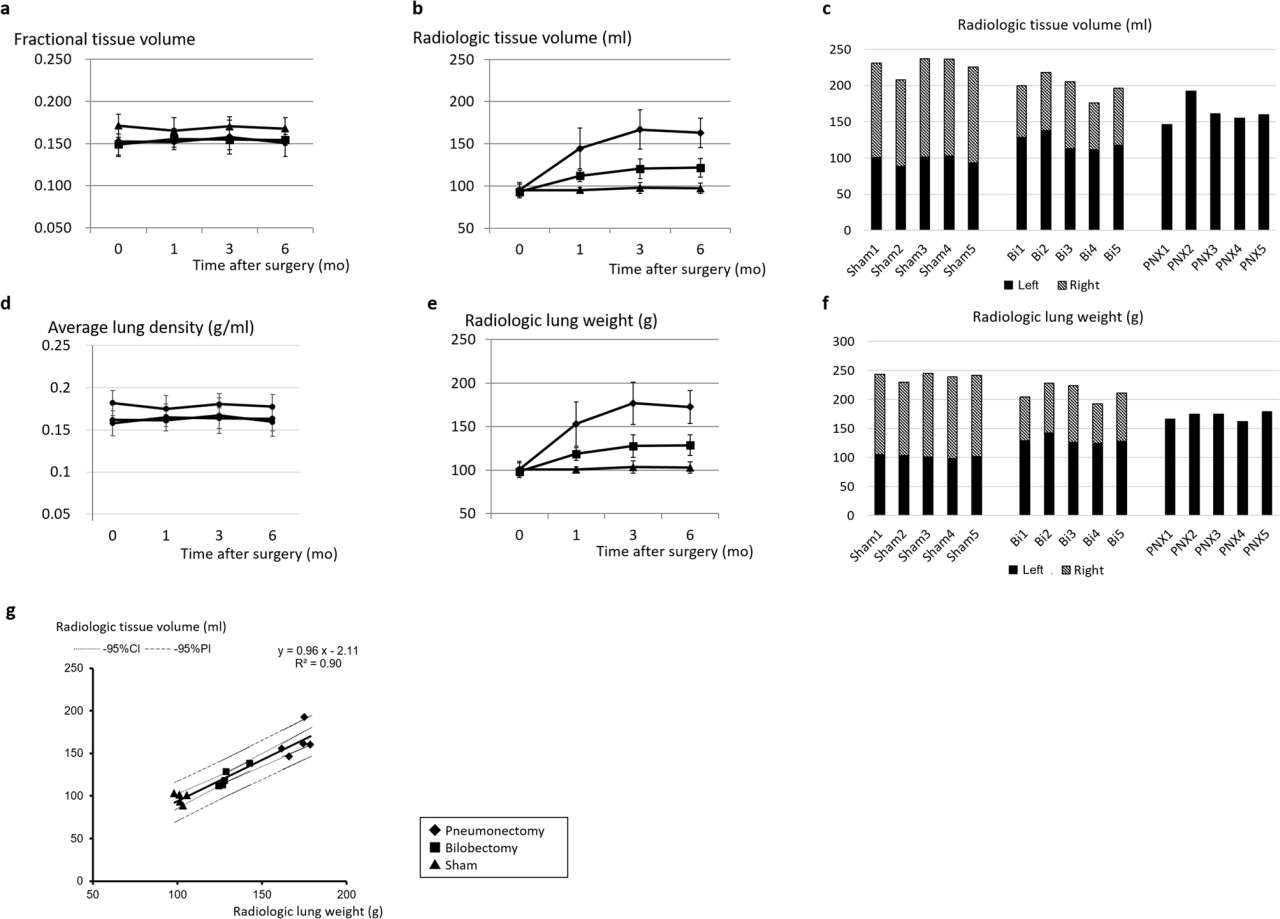
(**a**,**b**) The value changes of the fractional tissue volume (**a**) and of the radiologic tissue volume (**b**). (**c**) The values of radiologic tissue volume of the left and right lungs in each canine at 6 months after surgery. (**d**,**e**) The value changes of the average lung density (**d**) and of the radiologic lung weight (**e**). (**f**) The values of radiologic lung weight of the left and right lungs in each canine at 6 months after surgery. (**g**) Correlation between radiologic tissue volume and radiologic lung weight. Bi: Bilobectomy, PNX: Pneumonectomy.

Figure 2a and b exhibit the value changes of the fractional tissue volume and the radiologic tissue volume, respectively.

The change rates of fractional tissue volume, reflecting the density of lung tissue, were 98.8 ± 2.2% in the Pneumonectomy group, 106.3 ± 7.0% in the Bilobectomy group, and 98.2 ± 7.3% in the Sham group at 6 months post-surgery. The fractional tissue volume showed a change rate of 6.9% which was significantly lower than the 32.5% change rate in the lung volume (*p* = 0.0025, Fig. 2a).

The values of the radiologic tissue volume at 6 months post-surgery in the Sham, Bilobectomy, and Pneumonectomy groups were 97.2 ± 6.1 ml, 121.7 ± 11.7 ml, and 163.0 ± 17.4 ml, and the respective rates of increase were 2.4 ± 2.6%, 30.7 ± 7.4%, and 71.5 ± 7.4%, respectively. The rates of increase were significantly higher in the Pneumonectomy group than in the other two groups (*p* < 0.001, each, Fig. 2b), and the rate of increase in the Bilobectomy group was significantly higher than the one found in the Sham group. These findings are consistent with our results pertaining to the radiologic tissue volume.

##### Radiologic lung weight

The values of radiologic lung weight showed the same tendencies as the values of the radiologic tissue volume (Fig. 2d,e). More specifically, radiologic lung weight values at 6 months after surgery were 102.9 ± 6.5 g, 128.6 ± 11.9 g, and 172.5 ± 18.6 g in the Sham, Bilobectomy, and Pneumonectomy groups, respectively. Furthermore, the rates of increase in the postoperative 6 months were 2.0 ± 2.7%, 30.3 ± 7.2%, and 71.3 ± 7.0%, respectively. The rates of increase were significantly higher in the Pneumonectomy group compared to the other two groups (*p* < 0.001), and the rates of increase in the Bilobectomy group was significantly higher than the rate in the Sham group (*p* < 0.001). They showed the same tendencies with radiologic tissue volume. For comparison, the values of radiologic tissue volume and radiologic lung weight of the left and right lungs in each canine 6 months after surgery are presented in Fig. 2c and f.

##### Correlation between radiologic tissue volume and radiologic lung weight

There was a strong correlation between radiologic tissue volume and radiologic lung weight (correlation coefficient = 0.929, 95% CI 0.795–0.977, *p* < 0.001, Fig. 2g).

### The histologic evaluations of the residual lungs after lung resections

#### The anatomical lung volume of the fixed residual lungs at 6 months after surgery

**Figure 3:**
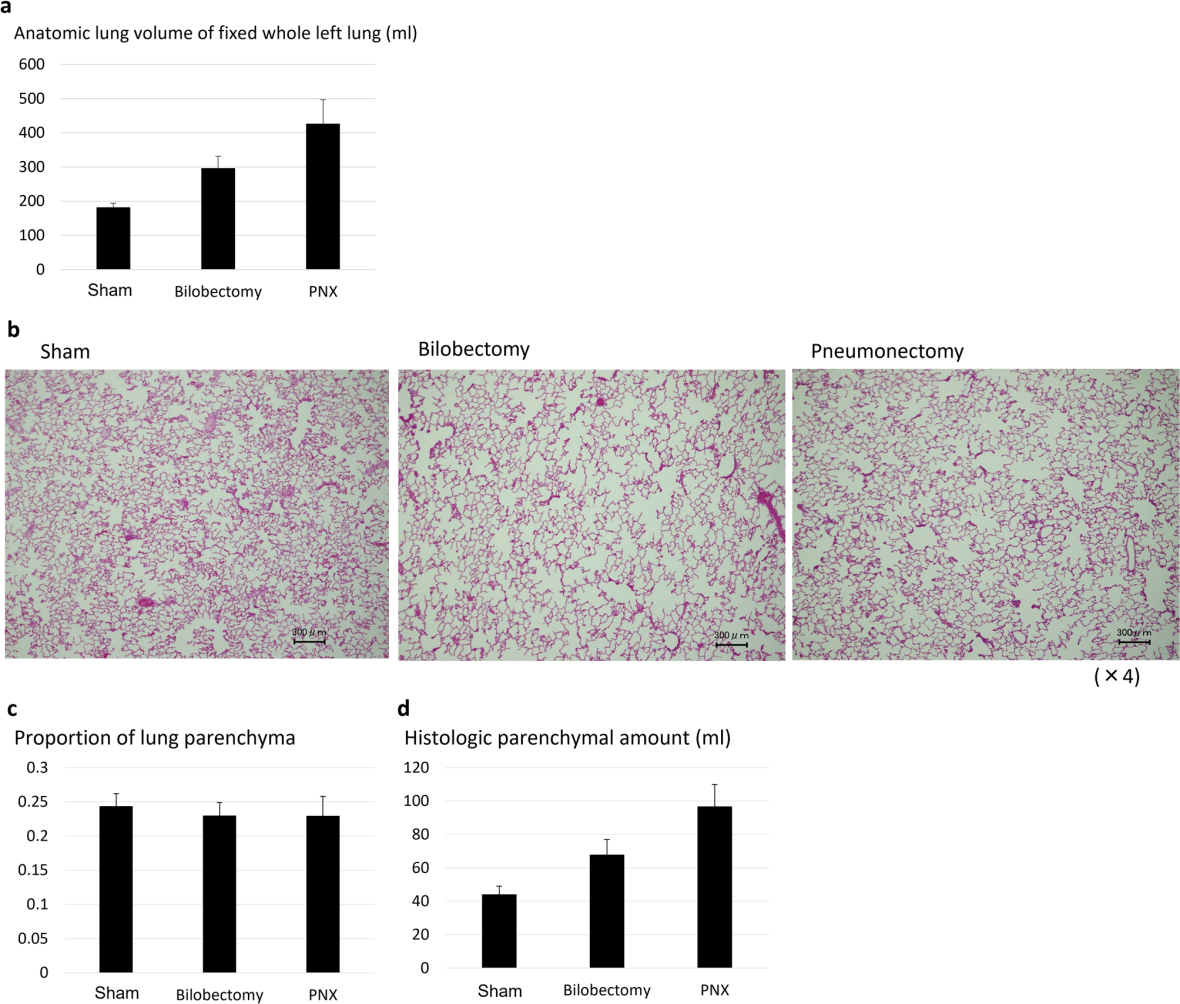
(**a**) The bar graph shows anatomical lung volume of the fixed left lungs 6 months after surgery of the Sham, Bilobectomy, and Pneumonectomy groups. (**b**) The representative histologic findings of residual left lungs of the Sham, Bilobectomy, and Pneumonectomy groups. Scale bar: 300 μm. (**c**,**d**) The bar graphs of the proportions of lung parenchyma (**c**) and the histologic parenchymal amounts (**d**) of the Sham, Bilobectomy, and Pneumonectomy groups. PNX: pneumonectomy.

Anatomical lung volume of the fixed left lungs at 6 months after surgery of the Sham, Bilobectomy, and Pneumonectomy groups were 180.6 ± 13.1 ml, 295.4 ± 36.0 ml, and 425.4 ± 71.6 ml, respectively (Fig. 3a). The lungs of the Pneumonectomy group showed greater anatomical volumes than those of the Bilobectomy and Sham groups (Pneumonectomy vs Bilobectomy: *p* = 0.002, Pneumonectomy vs Sham: *p* < 0.001), and the Bilobectomy group had greater anatomical volumes compared to the Sham group (*p* = 0.006).

#### Histologic lung parenchymal amount

Representative histologic findings of the residual left lungs of the Sham, Bilobectomy, and Pneumonectomy groups are shown in Fig. 3b. No apparent alveolar hyperinflation was observed in the Bilobectomy and Pneumonectomy groups despite the large increases in anatomical lung volume (Fig. 3b,c). There was not obvious vascular dilation in the alveolar region of the Bilobectomy and Pneumonectomy groups. The histologic parenchymal amounts of the Sham, Bilobectomy, and Pneumonectomy groups were 44.0 ± 5.0 ml, 67.6 ± 9.3 ml, and 96.5 ± 13.3 ml, respectively (Fig. 3d). The Pneumonectomy group exhibited greater histologic parenchymal amount than the Bilobectomy and Sham groups (Pneumonectomy vs Bilobectomy: *p* = 0.002, Pneumonectomy vs Sham: *p* < 0.001), and the Bilobectomy group had larger histologic parenchymal amount compared to the Sham group (*p* = 0.007).

### Comparison between histologic and radiologic results

**Figure 4:**
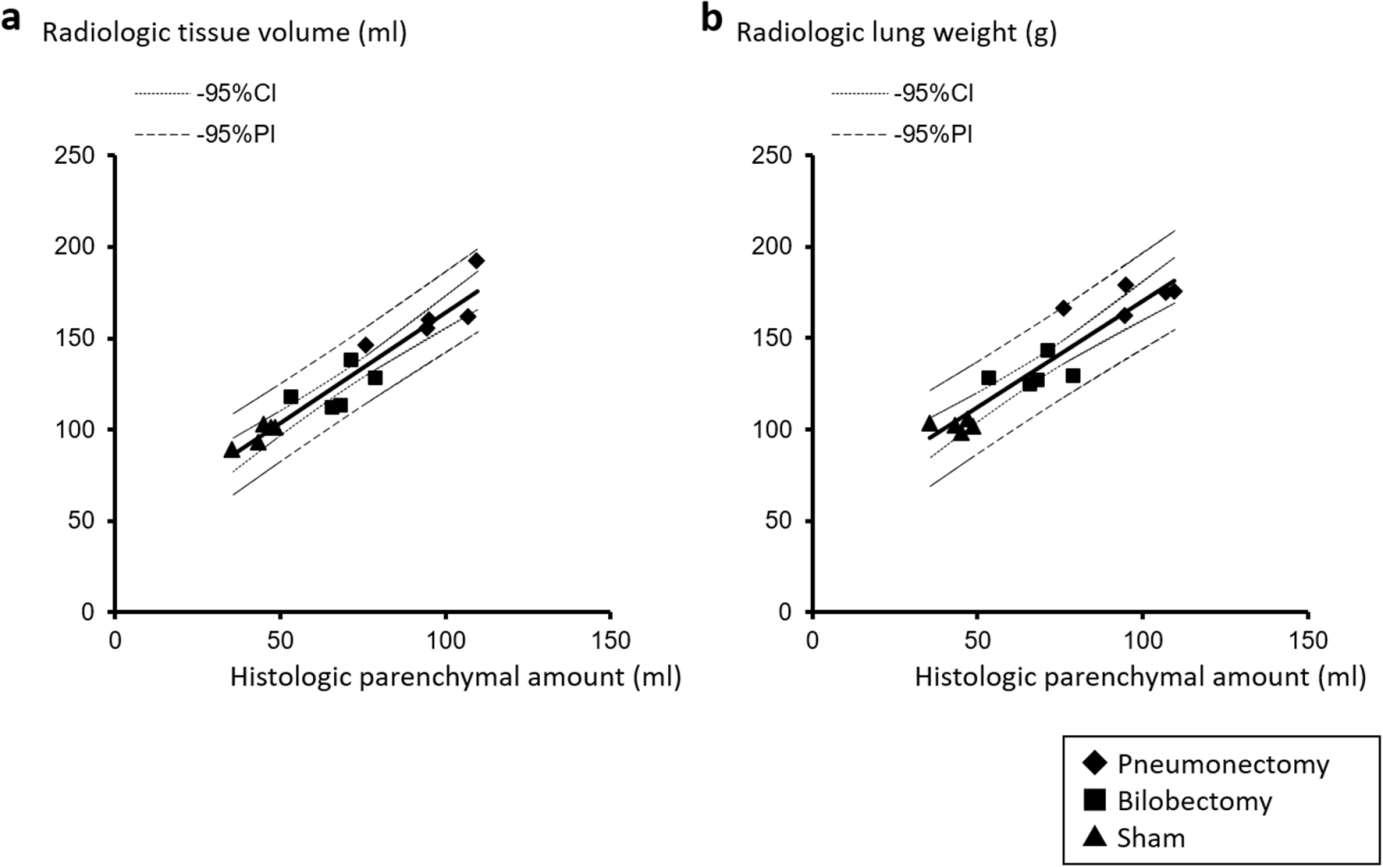
Correlation of the values of lung parenchymal amount measured by computed tomography analysis (**a**: radiologic tissue volume, **b**: radiologic lung weight) and by histologic morphometry.

Both radiologic tissue volume and radiologic lung weight were significantly positively correlated with histologic parenchymal amounts (correlation coefficient = 0.955 and 0.934, respectively, and *p* < 0.001 each, Fig. 4).

## Discussion

This study is the first report that assessed the validity of performing CT evaluations regarding qualitative alteration in the residual lungs after lung resection in a large animal model. Our results demonstrate that the residual lungs demonstrate compensatory lung growth in the relatively young canine model after major lung resections. Also, it is shown that CT can provide efficient information that reflects the amount of lung parenchyma following major lung resections. The previously reported two calculated values regarding “radiologic tissue volume”^8–10^ and “radiologic lung weight”^11^, were significantly correlated with the histologic parenchymal amount and worked similarly as effective evaluation methods of compensatory lung growth. Our results support the validity of the radiologic evaluation using CT data.

One of the largest issues in clinical lung growth studies is that there is no standard evaluation method to assess qualitative lung changes. Radiologic analysis using CT data may be ideal for compensatory lung growth assessment for several reasons. First, CT examination is a minimally invasive examination of lung structures even for postoperative patients. Therefore, histologic assessment of tissue samples obtained from invasive lung biopsies can be avoided. Second, CT examination evaluates the entire lung and compensatory lung growth is considered to provide heterogeneity due to regional lung expansion. CT data cover the entire area of the lungs, and thus have the capacity to evaluate this region in a more precise manner compared to a biopsy. Third, CT analysis is an objective and highly reproducible assessment between examiners, whereas spirometry often provides inaccurate results, especially when it comes to postoperative patients. Furthermore, various CT examination techniques have been widely used in the field of thoracic surgery such as CT volumetry and three-dimensional CT angiography^13,14^.

Radiologic tissue volume and radiologic lung weight were similarly correlated with the histologic parenchymal amount in compensatory lung growth. The acquired values primarily consist of two factors: lung volume and parenchymal proportion of alveoli. CT number, the value relating radiation absorption in one voxel, is the most important information obtained from CT images^7^. The CT number of air is set as − 1000 HU and the number of water as 0 HU, so that the CT number is expected to correlate with the fraction of parenchymal amount of lung composed of air and soft tissue such as alveoli, bronchioles, and vessels based on the predicted linear relationship between CT number and actual density. Radiologic tissue volume presents the volume of lung tissue excluding air in the lungs from the measured CT values of the lungs, air, and soft tissue. Radiologic lung weight is the value calculated from the hypothetical lung density, assuming that the density of air is 0 g/ml and the density of water is about 1 g/ml and that the density and CT value are proportional. The formulas for these values are very similar, and they were similarly effective evaluation methods of compensatory lung growth. It is important to note that radiologic lung weight, which we proposed in 2013, has a conceptual unit of g/ml, but there is no direct link between CT values and density. In fact, there was a large discrepancy between the radiologic lung weight and the actual mass measured in the preliminary experiment.

We employed ImageJ software^15^ for histologic lung morphometry, and our preliminary study demonstrates a strong correlation with the conventional point counting method (Supplemental material 1). The conventional point counting method is still used to estimate the volume fractions of alveolar septa. In fact, our lung parenchymal proportion measurements of the entire area may be more objective and easy-to-perform compared to conventional measurements.

The pneumonectomy model has played a pivotal role in the characterization of the physiological and structural adaptation of the lung to the loss of functional gas-exchange units observed in clinical lung resections and in a number of chronic destructive lung diseases^16^. Hsia et al*.* reported that right pneumonectomy (removing 55–58% of total lung) elicited the growth of new acinar tissue in the remaining lung^17^, however, left pneumonectomy (removing 42–45%) increased the remodeling of the remaining lung structure in canine^18^. In this study, we show that both right pneumonectomy and resection of lower and cardiac lobes, which removes only 32% of total lung, can promote neo-alveolarization. The amount of lung parenchyma increased, and the proportion of parenchyma was higher than the predicted values based on the anatomical lung volume increase ratio. Our data are consistent with a significant increase in alveolar number in canine lungs. This is perhaps due to the different types and ages of the dogs used and due to different surgical procedures performed such as the anterior mediastinal dissection that facilitates midline shift. Our results indicate the possibility that clinical lobectomy could elicit neo-alveolarization. Lung resections are a standard treatment for a number of diseases. However, several patients are unable to undergo this procedure due to poor or marginal respiratory function. Consequently, our findings may be applied to clinical practice because of the usage of mammalian young adults that did not grow in height.

There are several limitations in this study. CT examination was performed in the strictly conditioned situation. Pulmonary infiltrates such as in cases of pneumonia can undermine the accuracy of this modality in clinical practice. Furthermore, we did not perform lung biopsies during the postoperative period so as not to promote any post-procedural complications. Therefore, we could not evaluate potential histologic changes, including molecular events that may have occurred immediately after surgery. After lung resection, the blood flow into residual lung lobes increases. Large vessels are excluded in both radiologic and histologic analyses, but small vessels in the alveolar region may affect the results.

In conclusion, the histological examinations performed verify that the increase in radiologic tissue volume and radiologic lung weight reflect compensatory lung growth following lung resection. Radiologic assessment using CT data can be a very promising clinical method to evaluate postoperative compensatory lung growth.

## Methods

### Animals

Male beagle dogs aged 1 year and 10–11 months and weighing 9–12 kg were used in this study. This study was carried out in compliance with the ARRIVE guidelines (http://www.nc3rs.org.uk/page.asp?id=1357). All animals received care in compliance with the Principles of Laboratory Animal Care (National Society for Medical Research) and the Guide for the Care and Use of Laboratory Animals (National Institutes of Health Publication No. 86-23, revised 1996, Bethesda, MD). All surgical procedures were performed by Japanese board-certified thoracic surgeons in accordance with the Guide for the Care and Use of Laboratory Animals published by the National Institutes of Health (NIH Publication No. 85-23, revised 1985). The experimental protocol was approved by the Animal Experimental Committee of Kyoto University.

### Study design

The animals were randomly assigned into one of the following three groups: (1) the Sham group, where only right thoracotomies were performed, (2) the Bilobectomy group, where only resections of the right lower and cardiac lobes were performed, and (3) the Pneumonectomy group, where only right pneumonectomies were performed (*n* = 5 in each group). The mean ages of the Sham, Bilobectomy, and Pneumonectomy groups at the time of surgery were 22.2 ± 0.4 months, 22.6 ± 0.5 months, and 22.0 ± 0.0 months, respectively. The mean body weights of each group were 10.4 ± 0.2 kg, 10.5 ± 1.3 kg, and 10.2 ± 1.0 kg, respectively. There was no difference between three groups in the ages or body weights.

**Figure 5:**
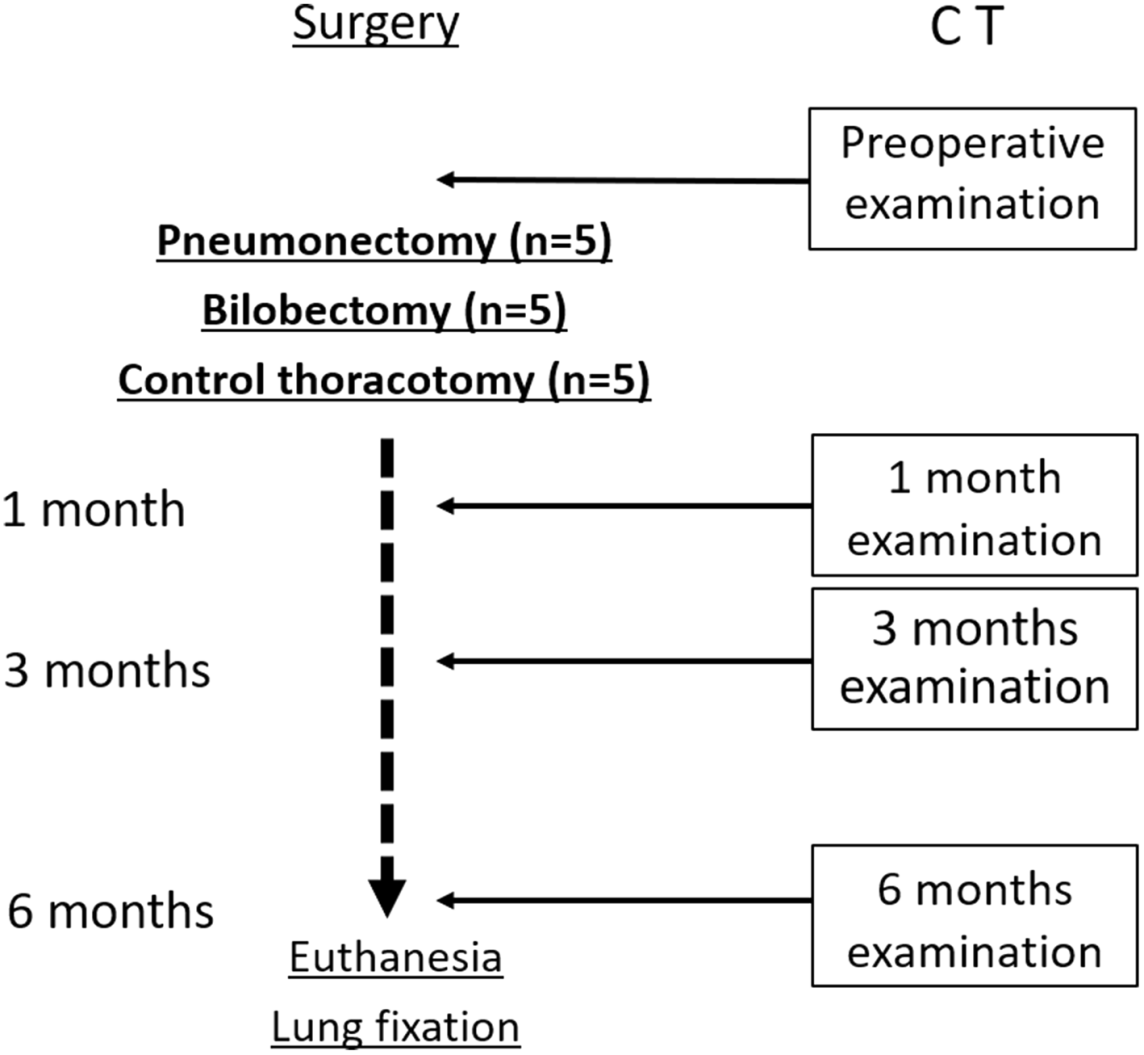
A diagram of the experimental protocol. Chest computed tomography (CT) acquisition to all animals preceded surgical procedures in order to establish a baseline. We performed control right thoracotomy, right lower and cardiac lobes, or right pneumonectomy to the animals included in this study (n = 5 in each group). CT images were also taken at the postoperative time-points of 1, 3, and 6 months after surgery. Following 6 months of postoperative acquisition, the animals were euthanized, and their lung-heart blocks were retrieved.

Chest CT examination to all animals preceded surgical procedures in order to establish a baseline. CT images were also taken at the postoperative time-points of 1, 3, and 6 months after surgery. Following 6 months of postoperative acquisition, all animals were euthanized, and the lung-heart blocks were procured. Radiologic evaluations and histological findings at the postoperative month six were then compared to assess the validity of radiologic evaluations (Fig. 5).

### CT acquisition

All examinations were performed using a multi-detector CT scanner (Aquilion 16; Canon Medical Systems, Tochigi, Japan) and the images were archived in Digital Imaging and Communications in Medicine format. Axial CT images were taken with 1-mm thick slices. The CT acquisition protocol is presented in Table 1.

**Table 1:** The protocol of computed tomography acquisition.

CT acquisition setting	
Tube potential	120 kVp
Tube current	Auto exposure control (50–300 mA)
Gantry rotation time	0.5 s
Primary collimation	16 row × 1 mm
Tube current–time modulation	Use
Acquisition mode	Helical
Helical pitch	0.9375
Image reconstruction	FBP
Reconstruction kernel	FC13
Matrix size	512 × 512
Image slice thickness	1 mm
Image overlap / gap	1 mm

Animals were fasted overnight, and they were anesthetized with a subcutaneous administration of 15 mg/kg ketamine hydrochloride, 7 mg/kg xylazine, and 0.5 mg/body atropine sulfate. Subsequently, they were intubated with an 8.5-mm cuffed endotracheal tube and were mechanically ventilated. A 500-mg dose of ampicillin was subcutaneously injected and general anesthesia was maintained with inhalational sevoflurane. CT examinations were performed in the supine position, and a pressure-limited respirator was used to maintain the animals’ breath at transpulmonary pressure of 20 cm H_2_O (Evita 4; Drӓger, Lubeck, Germany).

### Operation procedure

After the preoperative CT acquisition, the right 5th intercostal thoracotomy was performed in the lateral decubitus position. In the Bilobectomy and Pneumonectomy groups, resection of right lower and cardiac lobes and right pneumonectomy were performed, respectively; pulmonary arteries and veins were dissected and ligated with 3–0 silk sutures and bronchus with No. 2 silk suture. Anterior mediastinal tissue was dissected to facilitate postoperative midline shift in all groups. The bony thorax was closed with No. 2 polyglactin stiches (Vicryl, Ethicon, Inc., NJ, USA) and the muscles and skin were closed in layers with 3–0 polyglactin stiches. An additional 500-mg dose of ampicillin was injected subcutaneously at the end of surgery.

### Radiologic evaluation

CT images at transpulmonary pressure of 20 cm H_2_O were transferred to the AZE Virtual Place Lexus workstation (Canon Medical Systems, Tochigi, Japan). The workstation automatically executed the segmentation of the left lung and calculated the volume (ml) and the average CT number (HU) of lung. The trachea and the next three generations of large conducting airways were excluded in the lung segmentation. Then, manual measurement of the CT number of tracheal air and skeletal muscle was performed to determine the estimated air and soft tissue CT number, respectively. The estimated air CT number was calculated by averaging the CT number of three separate regions of the tracheal lumen in each animal. The estimated air-free lung tissue CT number, i.e., estimated soft tissue CT number, was calculated by averaging the CT number of the infraspinatus, supraspinatus, and pectoralis muscles at the level of the carina.

#### Radiologic tissue volume

The fractional tissue volume and the radiologic tissue volume were calculated with the average CT numbers of the lung, lung volume, and estimated air density and estimated soft tissue density^8–10,19,20^. The fractional tissue volume, which represents a tissue density of lung tissue, was determined as follows:

$$ {\text{Fractional\;tissue\;volume}} = \frac{{{\text{average\;CT\;number\;of\;lung }}{-}{\text{estimated\;air\;number}}}}{{{\text{estimated\;soft\;tissue\;number }}{-}{\text{estimated\;air\;number}}}} $$

In contrast, the CT-derived radiologic tissue volume (ml), which represents the amount of lung tissue, was determined as follows:

$$ {\text{Radiologic\;tissue\;volume}} = {\text{radiologic\;lung\;volume}} \times {\text{fractional\;tissue\;volume}} $$

#### Radiologic lung weight

Radiologic lung weight was calculated with the modified protocol that was described in a previous report^11^, in which, the CT number of air of each image was used for standardization. The average radiologic lung density (g/ml), which represents an estimated tissue density of lung tissue, was determined as follows:

$$ {\text{Average\;radiologic\;lung\;density}} = \frac{{{\text{mean\;CT\;number }} - {\text{estimated\;air\;number}}}}{{\left| {{\text{estimated\;air\;number }}} \right|}} $$

Consequently, the radiologic lung weight (g), which represents the amount of lung tissue, was determined as follows:

$$ \begin{aligned} {\text{Radiologic\;lung\;weight}} & = {\text{radiologic\;lung\;volume}}\left( {{\text{ml}}} \right) \\ & \quad \times {\text{average\;radiologic\;lung\;density}}\left( {\text{g/ml}} \right) \\ \end{aligned} $$

### Histologic analysis

After CT acquisition 6 months after surgery, all animals were euthanized using an appropriate amount of anesthetic pentobarbital. The euthanized animals were then placed in the supine position to perform tracheostomy. When lungs collapsed following a small intercostal incision, we continued to instill 2.5% glutaraldehyde via airway at a 25 cm H_2_O pressure above the sternum in the thoracic cavity for one hour. After 1-h of in vivo tissue fixing, the lung was extracted from the thoracic cavity and was subsequently immersed in glutaraldehyde for 24 h. The pressure of airway instillation was set higher than the intratracheal pressure during CT acquisition to adjust for the shrinkage effect of the lung specimen in the tissue fixation process seen in our preliminary experiments.

The anatomical lung volume was then measured using the immersion method^21^, and it was defined as the average of the two saline volume displacement measurements.

Tissue blocks were sampled from five different parts from each left lung. The sampling parts were randomly selected and adopted for all lungs. The tissue blocks were then fixed again with formaldehyde and were stained with Hematoxylin and Eosin.

The histologic proportion of lung parenchyma was calculated using the ImageJ software^15,22^. In this process, we performed background subtraction to correct for uneven illuminated background, conversion to 8-bit image data and reconstruction in black and white images, and we finally measured the area of lung parenchyma (Supplemental material 2). A magnification 40 × was used for evaluation. The histologic lung parenchymal amount was defined as follows:

$${\text{Histologic\;parenchymal\;amount}} = {\text{anatomical\;lung\;volume}} \times \frac{{{\text{the\;area\;of\;lung\;parenchyma}}}}{{{\text{the\;whole\;area}}}}$$

### Statistical analysis

Statistical analyses were performed with EZR (Saitama Medical Center, Jichi Medical University), which is a graphical user interface for R (The R Foundation for Statistical Computing, version 2.13.0)^23^, and the acquired values were expressed as mean ± standard deviation. The Shapilo–Wilk test was used for testing normal distribution. All animal characteristics were analyzed using the Kruskal–Wallis test. All data were analyzed by using one-way ANOVA, and we then used the Tukey’s post-hoc test to compare the values between the three groups. Finally, the Pearson’s correlation coefficient was calculated in order to detect potential correlation between the two variables. *P* values less than 0.05 were considered statistically significant.

## Supplementary Information

Supplementary Information 1.

Supplementary Information 2.
