# Supplementary figures and images for: Radiologic evaluation of compensatory lung growth using computed tomography by comparison with histological data from a large animal model
Source: Sci Rep. 2022 Feb 15;12:2520. doi: 10.1038/s41598-022-06398-y (PMC8847356; doi:10.1038/s41598-022-06398-y)

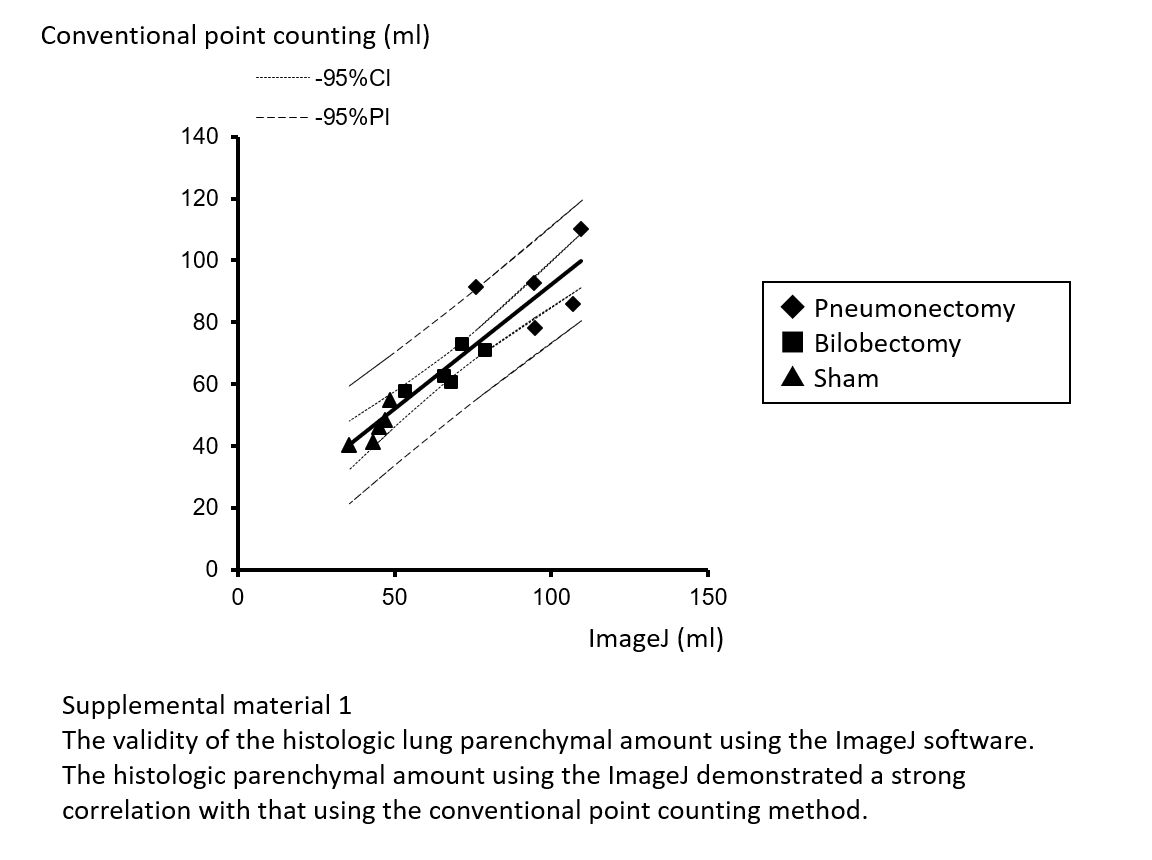

Supplement: Supplementary file 1 — Supplementary Information 1. [file 41598_2022_6398_MOESM1_ESM.tif]

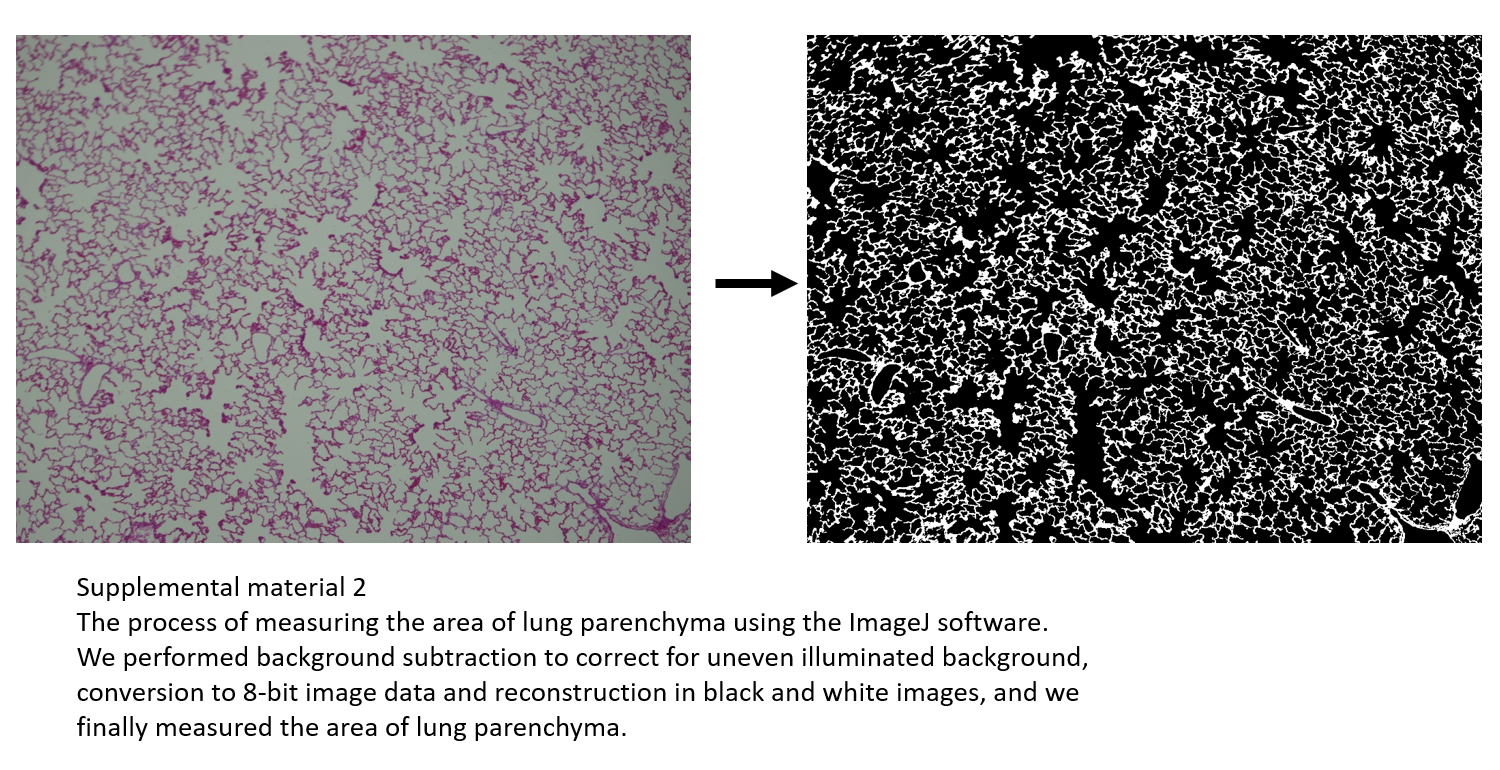

Supplement: Supplementary file 2 — Supplementary Information 2. [file 41598_2022_6398_MOESM2_ESM.tif]
